# Integrating care: the work of diabetes care technicians in an integrated care initiative

**DOI:** 10.1186/s12913-020-05109-5

**Published:** 2020-03-19

**Authors:** Christopher Bunn, Elissa Harwood, Kalsoom Akhter, David Simmons

**Affiliations:** 1 Institute of Health and Wellbeing, College of Social Sciences, University of Glasgow, Glasgow, G12 8RS UK; 2 Faculty of Health, Education, Medicine & Social Care, Anglia Ruskin University, Cambridge, CB1 1PT UK; 3 Department of Public Health and Primary Care, University of Cambridge, Cambridge, CB2 0SR UK; 4 Wolfson Diabetes and Endocrinology Clinic, Cambridge University Hospitals NHS Foundation Trust, Hills Road, Cambridge, CB2 0QQ UK; 5 School of Medicine, Western Sydney University, Campbelltown, NSW 2560 Australia

**Keywords:** Integrated care, Diabetes, Healthcare assistants, Qualitative

## Abstract

**Background:**

As diabetes prevalence rises world-wide, the arrangement of clinics and care packages is increasingly debated by health care professionals (HCPs), health service researchers, patient groups and policy makers. ‘Integrated care’, while representing a range of approaches, has been positioned as a promising solution with potential to benefit patients and health systems. This is particularly the case in rural populations which are often removed from centres of specialist care. The social arrangements within diabetes integrated care initiatives are understudied but are of particular importance to those implementing such initiatives. In this paper we explore the ‘work’ of integration through an analysis of the role played by Health Care Assistants (HCAs) who were specially trained in aspects of diabetes care and given the title ‘Diabetes Care Technician’ (DCT).

**Methods:**

Using thematic analysis of interview (*n* = 55) and observation data (*n* = 40), we look at: how the role of DCTs was understood by patients and other HCPs, as well as the DCTs; and explore what DCTs *did* within the integrated care initiative.

**Results:**

Our findings suggested that the DCTs saw their role as part of a hierarchy, providing links between members of the integrated team, and explaining and validating clinical decisions. Patients characterised DCTs as friends and advisors who provided continuity. Other HCPs perceived the DCTs as supportive, providing long-term monitoring and doing a different job to conventional HCAs. We found that DCTs had to navigate local terrain (social, ethical and physical), engage in significant conversation and negotiate treatment plans created through integrated care. The analysis suggests that relationships between patients and the DCTs were strong, had the quality of friendship and mitigated loneliness.

**Conclusions:**

DCTs played multidimensional roles in the integrated care initiative that required great social and emotional skill. Building friendships with patients was central to their work, which mitigated loneliness and facilitated the care they provided.

## Background

Diabetes is a serious and common chronic disease comprised of a complex interaction between genetics, physical, psycho-social, cultural and material lives. It is a major worldwide public health problem, affecting almost all populations in high-, middle- and low-income countries and high rates of diabetes-related morbidity and mortality are common [1]. The global population of people with type 2 diabetes is expected to increase to 439 million by 2030 [2]. In the UK, recent figures suggest that the population-adjusted prevalence of diabetes will grow from 6.6 to 7.4% between 2013 and 2035 [3]. This predicted growth in prevalence will increase costs to the healthcare system [4], and has potential to bring significant suffering to the lives of affected individuals, families and communities.

Both type 1 and type 2 diabetes are often destructive to a person’s body and life. The disease can affect multiple systems including: cardiovascular, renal, hepatic, ophthalmological, and nervous. As well as having physical effects, diabetes can also have detrimental impacts on mental health [5, 6], disrupt socio-cultural practices (particularly relating to eating) [7, 8], and can have a negative influence on post-diagnosis identities [9, 10]. Furthermore, people with both types of diabetes often experience social stigma relating to their condition and its treatment [11, 12]. Given the range of bio-psycho-social challenges faced by people living with diabetes, ideal health care benefits from contributions from a multiplicity of disciplines and roles: a range of medical doctors; nurses; dieticians; podiatrists; health care assistants; and psychologists. Integrating the care provided by this diverse range of healthcare professionals can be challenging and, if not carefully constructed, such services can fail to focus on the people they are serving [13].

Those who live in rural areas are often further away from centres of care and face additional costs and effort to reach appropriate treatment. In this paper we explore how health care assistants, with additional training to be “diabetes care technicians” (DCTs), contributed to the work of integration in a large-scale Diabetes Integrated Care Initiative (DICI) that was deployed and evaluated in rural East Anglia in the UK [14–16]. We ask two related research questions: how was the role understood by the DCTs, patients and other HCPs and what did the DCTs ‘do’ in the DICI?

### Integrated care, diabetes and rural settings

As pressures on diabetes services and health systems grow, allocating HCP time and healthcare resources most effectively and appropriately is a challenging task. A prominent paradigm in the response to this challenge is that of ‘integrated care’. In a 2016 working paper on integrated care, the World Health Organisation (WHO) took the position that the concept has multiple meanings:

… multiplicity stems from the polymorphous nature of the concept that has been applied from several disciplinary perspectives such as public administration, social science, psychology as well as differing professional points of view, such as clinical vs. managerial, holistic care vs. disease management and public health vs. long-term care. Integrated care is, therefore, associated with a wide range of different objectives [17].

Using scoping review methodology, researchers at the WHO constructed three broad operationalisations of ‘integrated care’. The first takes a ‘process-based’ position, which is often used by governments, and emphasises funding pathways, models of care, alignment of priorities and collaboration across sectors. The second, is a ‘user-led’ definition which foregrounds integration at the level of the person and seeks to achieve coordinated care which puts individuals in control. Finally, the WHO identify a ‘health system-based definition’, which focuses on promoting forms of care that cohere across the life course and are tailored to the multiple needs of a state population [17].

In the UK, integrated diabetes frameworks have been pursued for more than 15 years, and pilots of all three varieties identified by the WHO review have been implemented [18, 19]. These frameworks have promoted integrated care and are often focussed on ensuring that those living with diabetes receive an ‘annual review’ within primary care, during which complications can be detected and then referred on to relevant specialists for treatment, such as diabetologists, podiatrists and dieticians. Integrated healthcare records, while often controversial, are the cornerstones for establishing integrated care teams [20]. A London-based study implemented such a system of integrated record sharing, multi-disciplinary team meetings, collective governance and new financial arrangements to improve diabetes care and outcomes [21]. Evaluation of this study found significant improvements in cholesterol and blood pressure control, but not in glycaemic control. Internationally, however, an integrated approach to managing type 2 diabetes and depression via ‘integrated care managers’ has been shown to improve glycaemic control and improve mental health [22]. A review of 13 integrated Diabetes Care case studies around the world, including three from England, three from the USA, three from continental Europe and one each from Africa and Asia, suggested that while integrated diabetes care can improve metabolic control and reduce hospitalisation, governance arrangements were pivotal in ensuring concrete collaborative arrangements were in place [23].

Evidence relating to the implementation of integrated care in rural settings is considerably less developed than that relating to urban centres, but recognition of the need for good information has been present in the policy discourses of developed nations for more than a decade [24]. This reflects a general dearth of epidemiology that adequately addresses urban/rural differentials in health outcomes [25]. In the UK, a study in the Devon area suggested that the integrated models of care have potential to improve foot care for people with diabetes [26]. In the remote Scottish Highlands, an integrated care initiative was designed collaboratively by more than 300 health care professionals with the aim of creating a patient-centred service combining a multidisciplinary team with an integrated information system, but no evaluation has been published [27]. In the US, telemedicine, web-based services, helplines and community health advisors have been recognised as potential solutions to the challenges faced in rural diabetes care [28]. In South Africa, Distiller and colleagues have shown that a community-based integrated care programme, which followed a community-based capitation and risk-sharing model for both type 1 and type 2 patients, was able to achieve reductions in hospitalisations and produce significant improvements in HbA1c at both 1- and 5-year follow-up [29].

While integrated care for people living with diabetes has been the subject of much clinical research, less is known about the social relationships through which integrations of care are achieved. The effort required to integrate care for a patient is especially apparent in research that has addressed the experience of carers who strive to produce ‘seamless’ care for those they look after, by co-ordinating appointments, treatments and acting as the ‘link’ between disparate care providers [30]. Such research echoes wider sociological perspectives on life with chronic disease and reveals integration to be a form of ‘work’ which requires practical and emotional labour [31–33]. In formal integrations of care, healthcare systems aim to relieve carers and patients of the work of integration, but often face challenges relating to the siloed nature of existing provision and concomitant incentive structures [34]. Through our focus on DCTs, we seek to contribute to the study of the social relationships in integrated care initiatives by exploring their contribution to the *work of integration*.

## Methods

### The setting and the DICI

Launched in 2008, the DICI sought to integrate primary, community and secondary care in the two districts of East Cambridgeshire and Fenland (ECF) areas. The combined population of the area at the time of the study was 179,279 spread over 1197.7km^2^, giving a population density of ~ 150/km^2^ [35]. Prevalence of diabetes in the study area was 5.0%. No major hospital exists in either district which, together, span the catchment areas of five hospitals.

The initiative included: (a) organisational redesign, which established a network of local diabetes professionals; (b) providing specialist support for primary care providers through the allocation of a consultant, diabetes specialist nurse (DSN), diabetes specialist dietician and diabetes specialist podiatrist to each practice, as well as the provision of ‘virtual’ clinics in which advice on individual patients was given to primary care providers through the collective review of medical records and discussion; (c) increased patient, primary care and community education; and (d) increased care closer to home, including in multidisciplinary community-based clinics, increased access to diabetes specialist nursing, dietetic and podiatry clinics and increased home visiting by DCTs. A full description of the DICI can be found in [16]. In the terms set out by the WHO review, the DICI was a combination of all three ‘types’ of integrated care.

### The role of the DCTs

Within the DICI, the specific role of the DCT was to support patients: who had recently been asked to make significant changes to their medication (e.g. starting or changing insulin dose); who found it difficult to get to care providers (due to impairment, social and/or economic related reasons); and/or had a history of non-attendance. DCTs were provided with protocols for visits to patients who had recently started insulin treatment. DCTs were also trained in providing advice relating to testing blood glucose, tablet-based treatments, non-diabetic medications, caring for the feet, diet, physical activity, and general wellbeing. While DCTs were not allocated a fixed number of patients, the DICI service saw 521 people and conducted 1362 home visits in a 6-month period in 2009–2010 [36].

### Data collection and analysis

The work presented here is based on an 18-month qualitative study among those with known diabetes in ECF areas. Twenty-one male and female patients with diabetes who were receiving primary, secondary and/or community diabetes care were identified by the clinical teams from local health services. The patients were purposively selected by age, gender, and the following criteria: having diabetes for more than 5 years and a recent HbA1c test of 9 + % (75 mmol/mol), or any hospitalisation in the previous 12 months. Direct observations of patient consultations with doctors, dieticians, nurses and HCAs were carried out by EH and CB (*n* = 40). DCT home visits were also observed by EH (*n* = 22). Observations took the form of non-participant observation, fieldnotes were taken during these observations and the fieldnotes were subsequently written up as ‘thick descriptions’ [37]. Semi-structured interviews with participating patients were conducted at baseline (*n* = 21) and at between 6 and 12 months later (*n* = 17) by EH and CB. HCPs (*n* = 17) also participated in semi-structured interviews conducted by EH and CB (see Table 1). All observations and interviews were conducted by experienced social scientists, EH and CB. Each interview lasted between 25 and 90 min and was digitally recorded. Patients were interviewed at home whenever possible in order to ensure they felt at ease and were able to comment freely about their experiences. All interviews were transcribed verbatim. Topic guides used in the research can be found in Supplementary File 1.

**Table 1:** Age, sex and roles of interview participants

Patient Participants	N	Age (mean, range)
Male patients	12	57.4 (38–76)
Female patients	9	53.6 (28–80)
**HCP Participants**	**N**	**Sex**
Diabetes care technicians	2	0 (M)/2 (F)
Nurses	5	0 (M)/5 (F)
Hospital-based doctors	3	3 (M)/0 (F)
General Practitioners	2	1 (M)/1 (F)
Dieticians	2	0 (M)/2 (F)
Podiatrists	3	0 (M)/3 (F)

Following the structure of the two research questions, data were analysed thematically by CB, EH, KA [38], with each researcher coding data inductively before establishing a codebook, which was subsequently applied. Interview data were analysed to establish the different ways in which DCTs, patients and other HCPs described the role of the DCT. This analysis produced the following themes (interview group in brackets): explaining clinical decisions, hierarchy, linking, validation/reassurance (DCTs); advisors, continuity, friendship (patients); differences to other HCAs, long-term monitoring, support (HCPs). In relation to the second question, observation data were collected together under the following thematic headings (codes in brackets): navigation (avoiding stigma, cultural sensitivities, privacy, rural terrain); conversation (biography, catching up, family, medication, social lives); and negotiating integration (goals and aspirations, making ‘deals’, social and emotional labour, treatment choices).

Written informed consent was gained from participants before being admitted to the study. In addition, patient and HCP consents were gained orally before observations and interviews. All audio-recordings were de-identified for analysis. Ethical approval for the study was received from the National Research Ethics Service Committee, East of England (June 2011: 11/EE/0148).

## Results

### How is the role of the DCT understood?

In this section, we present an analysis of how DTCs, patients and other HCPs perceived the role and work of the DCTs.

#### The views of DCTs

DCTs generally narrated their role in *hierarchical* terms. One described her work as supporting nurses and dieticians, but did not mention doctors:

***CT01:****The role I play in diabetes service is I support the nurses and the dietician basically. I go out to patients … who have started insulin and I follow in sort of ten days later and we go through a protocol, depending on the patient I go back two or three times, or sometimes it’s only just the once or twice. And just go through a protocol of what’s needed, if the device is working, how much insulin are they on, blood sugars, checking things like that. But we also go through diet, exercise, tablets, other medications and just wellbeing really. So it’s a really supportive role to the nurses and the dietician.*

When asked about the wider network of care, DCTs saw their work as *linking* patients to nurses and doctors at local surgeries, and other members of the integrated care team, as well as family and friends. One DCT also spoke of the less visible people in the social network of care: administrative staff, neighbours, social workers and other patients.

DCTs described their role as one of providing *validation and reassurance* in relation to decisions made by doctors or nurses, particularly changes in treatment regimen.

***INT: Okay. So they might have been told at their practice or by someone else that they’re about to change things so they want another opinion?***

***CT01:****Yeah. Or they’re, because half the time I’m in there with them when I do change it but if DN03’s been in say for a visit or DN04, they’ll say “well they changed me, what do you think?” you know, and I’ll say “well I think they’ve changed that for the right reasons” and we’ll go through all the reasons why they’ve done that as well, so yeah. So they do, even though I can’t change medication they will involve you in that which is, you know, I suppose through it’s trust isn’t it? They trust you, yeah […] But I do think they see, they want you as kind of a friend, a lot of them, a lot of the elderly I think see you as like a daughter or something, or granddaughter […] Because they’re quite lonely I think, that’s what it is.*

Validation and reassurance relating to treatment decisions, in this account, is entangled with trusting, friendship or family-like relationships, which the DCT attributes to loneliness.

The DCTs also described their role as helping to *explain decisions* where information is either inaccessible or difficult to understand.

***CT02:****A lot of the time, where I work in the Town1 area, it’s often because people are dyslexic, often because they don’t trust their doctors’ practice because they use very big words that they don’t always understand and they don’t want to feel silly. And often because they’re housebound and can’t get out, so you going to them, they see that as you doing them a big favour and a lot of them would never go to the surgery, so okay, maybe the information has been there, but they haven’t been able to access it and a lot of my patients aren’t computer-literate.*

In this account, the DCT presents the role as one of translator or explainer of biomedical discourse, with the intention of helping patients understand the aspects of care that are opaque to them.

#### Patient views

Patient views of the DCTs were overwhelmingly positive and repeatedly framed relationships in the terms of *friendship*:

***P13:****CT01 is all I need really, well she can only do so much but on the other hand she’s somebody that I can make suggestions and she’ll say “well yes or no”, you know… Very relaxed because I can talk to CT01 about anything and we are friends, she feels the same way, you know.*

Alongside friendship, as P13 describes, the DCTs were also treated as *advisors* who could help a patient explore potential treatment options.

Alongside friendship, the DCTs also provided *continuity* when other HCPs moved onto new positions:

***P19:****CT01 came in one day and she said “DN04’s gone”, I said “so I hear”, one of the nurses or doctors or something told us […] And it really upset me that because I thought she was so nice and we got on so well. She could have told me the last time she was coming that I shan’t be coming again, you know, just to simply drop off like that is… But then I suppose it’s like everything else, a rat race, everybody for themselves. I don’t like that sort of attitude but there you go.*

Patient narration of the DCTs focussed on their roles as friends and maintainers of continuity. Not only did DCTs provide a space for talking about ‘anything’, they also offer familiarity at times when routines of care were altered.

#### The views of other DICI HCPs

During interviews, the majority of the DICI team mentioned the DCTs (or as Health Care Assistants (HCAs)) when asked to name those involved in DICI diabetes patient’s network of care. One nurse described the role of the DCT as delivering long-term *monitoring* and *support*:

***DN03:****And the Care Technicians as well, I mean they’re absolutely a great support, they will see patients after we’ve commenced them on insulin, following their protocols to offer on-going education and support as well. I mean they do an amazing job with patients, like podiatry advice and regular weighing as well, teaching people to test their blood with blood glucose meters.*

***INT: So quite practical sort of advice there?***

***DN03:****Yes, because as again they’re in a privileged position when they go in regularly and it’s continuing the on-going education.*

In this nurse’s account, the DCTs are positioned as both ‘a great support’ and as inhabiting a ‘privileged position’: they provide reinforcement and longer-term engagement than nursing staff can offer, and also build strong relationships by ‘go[ing] in regularly’.

Another perspective on the DCTs came from one of the registrars who worked in the DICI:

***DR04:****The team that I work with… so essentially it’s myself who’s the doctor, obviously the consultant, DR02, we’ve got diabetes specialist nurses, and, with me, those who primarily work in the community, then we’ve got community dieticians and also community health care assistants [DCTs] as well. The health care assistants in the community are, I feel, different to the ones in the hospital, um, certainly the ones that I work with have a bit more of a role in terms of follow-up and supporting patients to give them support between clinics.*

In this account, the registrar acknowledges and points to a *different* level of DCT/HCA engagement with patients than the equivalent hospital-based roles are afforded.

### What do DCTs do?

Analysis of observational data produced three themes through which we present the work done as part of the diabetes care technician role: *navigation*; *conversation;* and *negotiating integration*.

#### Navigation

Within the DICI, diabetes care was provided by a multi-disciplinary team in multiple settings (e.g. GP practices, community clinics, and the regional hospital). Patients who had recently commenced insulin treatment or changed their pill-based regimen, found it difficult to get to care providers (due to impairment, social and/or economic related reasons), or had a history of non-attendance at clinics, were visited by a diabetes care technician. The area covered by the DCTs offering this care necessitated a variety of forms of mobile working.

*CT01’s vehicle is her office for the day and her diary and mobile are out when I get in. She’s checking voicemails and trying to decipher a message. There isn’t particularly good reception, making the sound cut-out during the message.*

The difficulties of delivering care on-the-move in a rural setting, as exemplified by the lack of mobile phone reception in CT01’s mobile ‘office’, contrasts to the more predictable nature of a GP surgery or hospital. While a ‘static’ HCP may need to learn the hallways of a building, community-based professionals require knowledge of a wider (techno)geography, including where you can and cannot get mobile reception.

As well as a need to navigate the geography of mobile phone reception in the areas they operated in, the DCTs also have to traverse social terrain. For example, when a patient is not at home at the time of a scheduled appointment, some DCTs know where to ‘find’ their patients:

*This patient has a “lady-friend” (or ‘girlfriend’) who lives close-by and he usually has lunch there one day a week. He’s not at home when we arrive, but CT01 knows where he’ll be and pops to his lady-friend’s house a few doors down to see him. They walk back from the house together towards the patient’s house.*

In this observation note, we see that the DCT deploys knowledge of the patient’s social connections, which were shared freely in earlier appointments. While formal operating procedures may position such a maneuver as an invasion of privacy, the DCT made a tactful judgement based on knowledge shared in previous interactions.

While such judgements are not without risk, DCTs were observed to take privacy very seriously, noting that protecting patient confidentiality while working in the community can be a major challenge.

*[CT02] says the care technicians arrange appointments to ensure visits within close proximity have some time between them, so patients are less likely to see them entering a neighbour’s house. Another aspect of protecting patient anonymity is their lack of uniforms. By entering the home without a nurse’s uniform, they are less likely to be identified as a health care professional.*

From a logistical perspective, it is counterintuitive to visit patients in any other order besides the shortest route, but in communities where diabetes can be stigmatised (and patients blamed for lifestyle choices which are perceived to contribute to their condition), the DCTs try to prevent the possibility of such unintended exposures.

Observations of DCTs at work suggested that the community-based nature of their practice requires multiple competencies that can be captured with the term ‘navigation’. Besides the physical geography of locations and routes, the role requires navigating technological, social and ethical spaces.

#### Conversation

Conversational exchanges between DCTs and patients play an important role in how diabetes-related matters are discussed in their interactions. Sometimes exchanges are very brief and other times lengthier. These exchanges cover topics including personal matters such as a patient’s family and pets, the people the patient is in regular contact with, and broader local topics such as a doctor’s new beard. Conversation between the DCTs and patients often begin with a ‘catch up’:

*DCT01 ‘catches-up’ with the patient, as she has done with all her patients. For about ten or fifteen minutes they chatter. They discuss the lady’s son-in-law who has been diagnosed with diabetes and how she is giving him advice on what to do, including jokingly scaring him about how awful it is. The conversation eventually turns to diabetes with DCT01 changing tack to the effect of ‘I suppose I better ask you about your diabetes, now we’ve righted all the wrongs with the world’ and DCT01 asks how she’s getting on with her medication and sugars. The patient is having no problems and the conversation soon returns to more general chatter. The patient gives DCT01 a marrow and some courgettes (from her vegetable patch) and gossip magazines she’s finished reading.*

Such conversations were typical in the observation data. Through regular and linked interactions, the DCT and their patients build a rapport such that family can be discussed, vegetables exchanged and the ‘wrongs’ of the ‘world’ can be ‘righted’.

Informal conversations during visits were not just trivial ‘chit-chat’. In the following encounter, the DCT sees a patient who has stopped taking their medication:

*Like old friends, they discuss the gardening and whether the lady’s son would be around to help. The lady seems annoyed with her son, a farmer, who hasn’t been around to see her recently. CT01 suggests he might be busy with the farm. After some polite chatter, CT01 broaches the subject of her diabetes and asks how her medication is. The lady says resolutely she’s stopped taking her medication. CT01 asks, with a hint of surprise in her voice, whether she’s told the doctor. “Why would I want to do that!?” CT01 asks which ones she’s stopped taking. “All of them!” CT01 starts to ‘scroll through’ all the medication she’s on. (CT01 seems to already know them all by memory.) CT01 asks if she can see which medications she’s on and goes to the kitchen to fetch boxes of pills. The lady says she has taken her blood pressure tablets “when she remembers” but stopped the others. CT01’s reaction is a combination of calm but concerned, with some use of light humour in response to the patient’s jokes. The patient seems not to want to discuss why she’s stopped taking her medication and tries to make a joke of everything CT01 says, often looking to me and winking as if she enjoys winding up CT01. CT01 allows her to joke for a bit, but then tries to steer the conversation towards a more serious tone, saying she’s worried and wants her to stay healthy. CT01 says she thinks it’s because she’s lonely, and the patient somewhat agrees, suggesting there’s nothing to do now. (This is the only ‘serious’ moment during our visit.) CT01 says she thinks it’s been difficult since her neighbours moved away and the patient says she’s considered getting another dog.*

While a strict biomedical perspective might see this conversation as a case of ‘non-compliance’ or failure to ‘adhere’, there are clearly other issues going on for the patient that influence her decision to stop taking her medication: frustration with a son; a need for humour; and the loss of the social contact provided by friendship with established neighbours. Through such exchanges, the DCTs are able to both support their patients as they narrate their lives, while at the same time addressing the agenda of diabetes self-management that is the formal basis of their interactions. Without CT01’s careful negotiation of the multiple concerns that are ‘live’ for the patient, the issue of medication taking may remain concealed.

The DCTs observed in the DICI invested time in understanding their patients’ lives, gathering detailed information about social networks, interests and concerns. While medical histories are vital for good care, they can be complemented by personal histories, often in ways that augment medical care:

*[CT02] had been visiting that patient with one of the nurses on the team and it took a few visits but they realised she was sleeping in the living room. She was getting up at 3am to do housework, making her sugar levels erratic. At first they tried to encourage her to stay in bed when she woke up. They eventually found out she had been a shiftworker and changed her doses to mimic this pattern and things had improved since then.*

The conversations between DCTs and their patients provide the patients with time to uncover biographical information that can inform care, such as past work routines or shifts in social engagement (absent sons and departed neighbours). These exchanges also offer the chance for DCTs to maintain relationships with those who have no current problems with their diabetes control, but may in the future.

#### Negotiating integration

Observational data suggest that one of the most significant aspects of DCT practice within the DICI was the construction of ‘bridges’ between patient and HCP perspectives on treatment.

*As we enter the house, we walk through to a large sitting room containing a settee where the patient is sat, surrounded by blankets and pillows. She looks unwell, ‘full of cold’. CT02 asks after her health and the patient says she is sick and feels awful. CT02 asks if she’s been to the doctor. Patient: ‘Why would I do that? They’ll just give me an antibiotic which will give me cystitis.’ CT02 pleads with her to go. The patient resists. Patient: “I’m not seeing a doctor”. CT02 asks after her children and what they think about her not seeing the doctor. The patient looks knowingly at CT02, implying they have discussed the children previously. I imagine the children must worry about their mother. CT02 strikes a ‘deal’ with her asking her to see the doctor if she’s not feeling better in two days, after some good rest. The patient reluctantly agrees.*

This exchange illustrates the resistance some patients exhibit towards HCPs. The patient believes the only help the doctor can offer would be antibiotics, which she feels will cause her additional pain (cystitis). Importantly, the patient’s disclosures – e.g. “why would I do that” – indicates that her judgment of ‘doctors’ does not extend to the DCT. Although harder to avoid than an appointment at a clinic, the patient does have the option of not answering the door, should they not wish to see the DCT. In this example, the DCT introduces leverage, in the form of the patient’s children, and uses this to negotiate a time point at which the patient will consult with her GP.

As well as negotiating the terms on which GPs are consulted, DCTs work with the wider goals and ambitions of the patient.

*CT01 says her diabetes is now well controlled but she just wants to lose a few more kilos so CT01 only visits once a month to weigh her. CT01 says she realises some of her patients are managing their diabetes better but they sometimes utilise her to help with other health issues like weight loss.*

Within the DICI, DCTs were able to assist with monitoring patients on insulin but were not authorised to advise on insulin adjustments and were required to check with DSNs before providing direction. The following example demonstrates a negotiation between a patient who trusted the DCT enough to inform her of a ‘hypo’ (hypoglycemia), but wasn’t willing to heed her advice regarding medication.

*After we enter the car, CT01 checks her phone and receives a message from a patient who has had a hypo. CT01 calls her back and finds out more information. The patient says she had a 3.7. CT01 asks how she treated the hypo and the patient says she had something to eat and drink. CT01 asks her to check her blood sugars now while she’s on the phone. CT01 waits and the patient says she is now 8.7. CT01 asks if she’s due to take her insulin soon, and the patient says yes. CT01 asks the patient to wait before giving her next insulin as she wants to check if she can bring it [insulin unit dose] down from 14 to 12. CT01 asks the patient to wait again before hanging up. CT01 calls one of her community DSN colleagues and says she’d like to change the patient’s insulin. The colleague agrees and CT01 hangs up to call the patient back. When she calls the patient back, the patient has already administered her insulin. CT01 looks exasperated very briefly, and asks her to change her next doses and to write down the new amounts. The patient is very grateful for CT01’s help and CT01 asks her to call again if she has any more lows.*

Negotiating the agendas of patients and their HCPs is particularly difficult when their trajectories are diametrically opposed. Recognising a want to die and rejection of treatment is not only challenging emotional work taken on by the DCT; for patients, these expressions are acts of defiance against the medical world. In the following encounter, the patient was over 90 years old and appeared very unwell.

*CT01 introduced me to the patient, describing where I was and touched his hand while she was talking to him. CT01 said his hand was cold and asked if he wanted his hand under the blanket. They discussed how he’d been feeling lately and where his son was. The son frequently visited the pub, which upset the man as he felt his son didn’t care about him. He mumbled something to the effect of it wouldn’t matter soon anyway. I realised this was the third patient CT01 had seen today who had seemed down with regards to their health. CT01 sat close to the patient and spoke in a loud voice, given his poor hearing. His diabetes was briefly discussed, but the visit seemed to revolve more around his general health and providing him with company.*

*[Outside, in the car] I ask CT01 about the fact that many patients seemed resolved about dying or their poor health: “Is that difficult to hear?” CT01 says a lot of the patients she sees are older and do have other health complications. She says she sometimes goes home and just sits quietly and thinks about what they’ve said.*

The DCTs that took part in the DICI were observed to undertake great social and emotional labour, particularly through their attempts to bridge the positionalities of patients and their healthcare professionals. In their daily labours, DCTs negotiate the terms on which glycaemic control (and related health conditions) is pursued, confront the likelihood of the immanent death of some patients, have to accept that their efforts to provide the best clinical guidance is sometimes ignored, but also have the opportunity to encourage and support patients in their goals e.g. losing weight.

## Discussion

In this paper we used interview and observation data to explore how the role of the DCTs was understood by actors in the DICI and what DCTs ‘do’ in a DICI. The DCTs understood their work in hierarchical terms, described their role as providing links to the work of other HCPs, and described explaining and validating the treatment direction set by other HCPs in the interdisciplinary team. Patient narratives suggested that many formed friendships with the DCTs that visited them, such that they trusted their advice on treatment suggestions, and provided continuity when other HCPs left the care team. Finally, the multi-disciplinary team described the role of the DCTs as providing the *long-term monitoring* and *support* that they were unable to, so much so that one member described their position as ‘privileged’. Significantly, DCTs were positioned as having a very different role to HCAs who work in hospitals, despite sharing ‘blueprint’ job descriptions and salary gradings.

The observational data we analysed suggest that the DCTs have to *navigate* terrain, people and ethical issues on a daily basis. Knowledge of the locations in which mobile phone signal is strong enough to communicate with patients and colleagues is vital for DCTs operating in a rural setting; the negotiation of local relationships (such as ‘lady friends’) requires DCTs to deploy tact based on trust, which is established over time; and the community-based nature of the work demands sensitivity to issues of privacy and stigma. Observations also suggest that a significant aspect of the DCT work involved *conversation* and that these conversations provided patients with a socio-cultural connection that was often lacking in their lives. These data also led us to conceptualise DCTs as people who helped patients to *negotiate* the concerns and routines of their lives alongside the suggestions, requests and demands of the *integrated* team charged with caring for them.

A key feature of the DCT role in this DICI relates to how they built and sustained friendships with their patients. In the accounts we have presented, it is clear that these friendships often mitigated loneliness, which is known to be linked to all-cause mortality [39, 40]. Many of the patients DCTs worked with were anxious about their children or neighbourhood environments. By regularly engaging with these narrated anxieties, the DCTs were able to help their patients express and negotiate social and emotional dimensions of their illnesses. This finding resonates with research that has championed nurse-led solutions to social isolation in chronic illness [41], and suggests that DCTs/HCAs might also contribute to patient care in this way.

While many studies of integrated care initiatives in diabetes focus on clinical outcomes, few have considered the social, practical and emotional dimensions of such ‘integrations’. Following the sociological literature on ‘illness work’, established by Corbin and Strauss [31], our study has used the positionality of the DCTs in the DICI implemented in East Cambridgeshire and Fenlands to explore the *work of integration*. This work included significant social and emotional labour [33], and echoes findings from studies of the role played by carers who manage multiple aspects of care [30]. Such work was enabled through by the social and cultural capital [32] that the DCTs established and utilised in their work, both of which were invaluable to the coherence and ‘integration’ of the DICI. This can be seen in data describing how DCTs learnt the social and cultural terrain of the communities they worked within to provide tactful care that patients could accept and rely on. Moreover, in how they built relationships that exceeded the clinical, through exchanging vegetables and stories relating to family, but which, at the same time, mediated clinically-relevant concerns and actions.

In a global context, our study echoes findings from studies of other community-based intermediary roles in diabetes self-management in disparate contexts. In the U.S. ‘Community Health Workers’ are characterised as ‘culture brokers’ that work within indigenous or minority communities to link patients to HCPs [42]. Review of this literature have suggested that community health workers can have significant and cost-effective impacts on patient outcomes [43]. In Latino communities, the relationship between ‘Promotoras’ and diabetes patients has been positioned as ‘almost kinship-level’ [44] and shown to have positive effects on HbA1c and diabetes knowledge after 6 months [45]. In Australia, Abbott and colleagues describe ‘Aboriginal Health Workers’ as partners of HCPs who help patients overcome cultural and communication issues in primary care settings [46], and including such a role can improve diabetes care in remote Aboriginal communities [47]. Gathering together findings such as these under the term ‘Community Health Workers’ (from the U.S.), Perry and colleagues conclude that these community-based roles can play a vital role in improving population health in low- middle- and high-income countries, especially when embedded within healthcare systems [48]. While not the subject of direct evaluation, our findings suggest that DCTs fit within this characterisation of community health workers; that through their navigation, conversation, mediation, and trust-building, they improved the care received by people living with diabetes in rural Cambridgeshire.

### Limitations

A limitation of this study is that the data we gathered through semi-structured interviews was from a purposive sample of patients, for which we had limited demographic data. This prevented us from systematically gathering narratives from across the socio-economic spectrum. A further limitation of the study is that no data was collected on the perspectives of support staff involved in the DICI. Data from interviews with receptionists and managers would have enhanced understanding of the DCTs and their work. Some may also argue that our choice not to measure inter-rater reliability is a limitation, but we note that this is a contested position to take which is conventionally opposed by the medical sociology community [49].

## Conclusions

The role of the DCT was set in a hierarchy, provided trusted links between patients the integrated team, explained and validated clinical decisions. The DCTs reassured patients, became their friends and advisors and offered continuity when staff changes took place. The wider integrated care team valued the support DCTs provided, the long-term monitoring they offered and noted that their work was substantially different from the work of other HCAs. The work of the DCTs required careful navigation skills, as they were required to traverse social, ethical and physical spaces; required significant conversational skill; and involved negating the care provided by the integrated service. In the rural setting of the DICI, our data suggest that the friendship offered by the DCTs mitigated loneliness and that their work has significant overlap with that undertaken by community health workers. Those designing integrated healthcare services for remote and vulnerable populations should consider including DCT-style roles.

## Supplementary information

**Additional file 1.**

## Data Availability

The datasets generated and/or analysed during the current study are not publicly available due to restrictions in the consent form, which was written and deployed before it was common practice for data to be open access, but are available from the corresponding author on reasonable request.

## Abbreviations

DCT: Diabetes care technician
DICI: Diabetes integrated care initiative
ECF: East Cambridgeshire and Fenland
HCA: Healthcare assistant
HCP: Healthcare professional
WHO: World Health Organization
