# Supplementary material for: Integrating care: the work of diabetes care technicians in an integrated care initiative
Source: BMC Health Serv Res. 2020 Mar 19;20:235. doi: 10.1186/s12913-020-05109-5 (PMC7082957; doi:10.1186/s12913-020-05109-5)
Supplement: Supplementary file 1 — Additional file 1. [file 12913_2020_5109_MOESM1_ESM.docx]

**Topic guide 1: healthcare professionals**

Please can you tell me about the role you play in diabetes care or services?

Please can tell me about how your role fits into the life of the patient with diabetes?

Are other professionals that help care for your patients?

Can you tell me about a time when a patient has felt least in control of their diabetes?

Can you tell me about a time when a patient has felt really in control of their diabetes?

Thinking about the last year or two, do you feel like anything has changed for you regarding your diabetes practice?

How do you understand ‘integrated care’ in a diabetes context?

How would you describe the relationships between primary, community and secondary care in diabetes?

If you could improve the way diabetes services are delivered what would you change?

**Topic guide 2: patient baseline**

Please can you tell me what an average day is like for you living with diabetes?

Please can you tell me about a time when you felt on top of or most in control of your diabetes?

Please can you tell me about a time when you felt on top of or most in control of your diabetes?

Thinking about the last year or two, do you feel like anything has changed for you when it comes to your diabetes care?

Does anyone help you with your diabetes? These people might be family members, friends or healthcare professionals.

Can you describe the last time you visited somewhere, or someone visited you, regarding your diabetes? What kind of experience was it?

If you could improve the way that health services help people with diabetes, what would you change?

**Topic guide 3: patient follow-up**

During your first interview, you talked about what your average day with diabetes is like. Please can you tell me how it has been since then; what is your average day now like?

Thinking about the last 12 months, please can you tell me about a time when you felt on top of or most in control of your diabetes?

Thinking about the last 12 months, please can you tell me about a time when you felt on top of or most in control of your diabetes?

Does anyone help you with your diabetes? These people might be family members, friends or healthcare professionals.

Can you describe the last time you visited somewhere, or someone visited you, regarding your diabetes? What kind of experience was it?

Thinking about the care you receive now and the care you used to receive, do you think anything has changed?

If you could improve the way that health services help people with diabetes, what would you change?
